# Health-related quality of life as associated with asthma control, psychological status and insomnia

**DOI:** 10.48101/ujms.v127.8967

**Published:** 2022-11-29

**Authors:** Anna Rask-Andersen, Mai Leander, Fredrik Sundbom, Erik Lampa, Anna Oudin, Bénédicte Leynaert, Cecilie Svanes, Thorarinn Gislason, Kjell Torén, Christer Janson

**Affiliations:** a Department of Medical Sciences, Occupational and Environmental Medicine, Uppsala University, Uppsala, Sweden; b Occupational and Environmental Medicine, School of Public Health, Sahlgrenska Academy, University of Gothenburg, Gothenburg, Sweden; c UCR-Uppsala Clinical Research Center, Uppsala University, Uppsala, Sweden; d Department of Public Health and Clinical Medicine, Occupational and Environmental Medicine, Umeå University, Umeå, Sweden; e Department of Laboratory Medicine, Occupational and Environmental Medicine, Lund University, Lund, Sweden; f Université Paris-Saclay, UVSQ, Univ. Paris-Sud, Inserm, Équipe d’Épidémiologie Respiratoire Intégrative, CESP, Villejuif, France; g Centre for International Health, Department of Global Public Health and Primary Care, University of Bergen, Bergen, Norway; h Faculty of Medicine, University of Iceland, Reykjavik, Iceland; i Department of Sleep, Landspítali – The National University Hospital of Iceland, Reykjavik, Iceland; j Department of Medical Sciences, Respiratory, Allergy and Sleep Research, Uppsala University, Uppsala, Sweden

**Keywords:** Health-related quality of life, asthma symptoms, anxiety, depression, insomnia

## Abstract

**Background:**

Asthma is associated not only with lower health-related quality of life (HRQL) but also with psychological health and insomnia. The aim of this study was to investigate associations between HRQL, asthma symptoms, psychological status and insomnia in adults from three Nordic countries.

**Methods:**

This study comprised 2,270 subjects aged 29–55 from Sweden, Iceland and Norway. HRQL was measured with the 36-Item Short Form Health Survey (SF-36). The physical (PCS) and mental health (MCS) component scores were calculated with higher values, indicating better health status. Symptoms of depression and anxiety were measured using the Hospital Anxiety and Depression Scale (HADS). Insomnia was assessed with the Basic Nordic Sleep Questionnaire. An asthma score consisting of a sum of the positive answers to five respiratory symptoms was used in the analysis. Spirometry and allergy tests were also performed.

**Results:**

High HADS and sleep disturbance scores were both related to a low PCS and MCS, respectively, after adjusting for confounders. High age and high body mass index (BMI) were associated with low scores on the PCS, whilst the opposite was found for the MCS. A higher asthma score was related to a low PCS. An interaction between the HADS and the asthma symptom score was observed for the PCS (*P* = 0.0002), where associations between psychological status and the PCS were more pronounced for individuals with more symptoms than for individuals without symptoms.

**Conclusions:**

In this study, we found that HRQL of life was independently related to the HADS, insomnia and asthma symptoms. Further prospective studies to identify the most efficient target for intervention in order to improve asthma control are needed.

## Introduction

Health-related quality of life (HRQL) has become an increasingly important aspect of outcome evaluations in health care and comprises an important outcome measurement based on individual perceptions of how a disease affects individuals in everyday life (1). Asthma control is regarded as the most important predictor of asthma-related quality of life. Other risk factors for reduced HRQL in asthma include female sex, young age, a low educational level (2–4), smoking, bronchial hyper-responsiveness (5) and asthma-related comorbidities such as chronic rhinosinusitis, obesity and depression (6–8). In a previous study, we found a strong association between respiratory symptoms and mental well-being (9). Several studies have reported a high prevalence of symptoms of anxiety and depression in asthma patients (3, 10–13). Furthermore, anxiety is not only overrepresented amongst asthmatics but also associated with more asthma symptoms, more frequent medical service trips and inadequate symptom perception (14).

Insomnia is another aspect of the psychological complexity in asthmatics, which has a high prevalence and a negative impact on HRQL (15, 16). Insomnia symptoms in asthma have most commonly been explained by poor asthma control, but the impact of asthma-related comorbidities, such as chronic rhinosinusitis, gastro-oesophageal reflux, obesity and symptoms of anxiety and depression, is significant (17, 18). Furthermore, the association between insomnia and asthma control is likely to be bi-directional, where poorer sleep quality may precede asthma symptoms (19). Similarly, insomnia may be an independent risk factor for depression (20).

The aim of this study was to investigate associations amongst HRQL, asthma symptoms, psychological status and insomnia in a sample of 2,270 subjects from centres in Sweden, Iceland and Norway, who participated in the ECRHS II (The European Community Respiratory Health Survey II).

## Material and methods

### Study design

**Figure 1:**
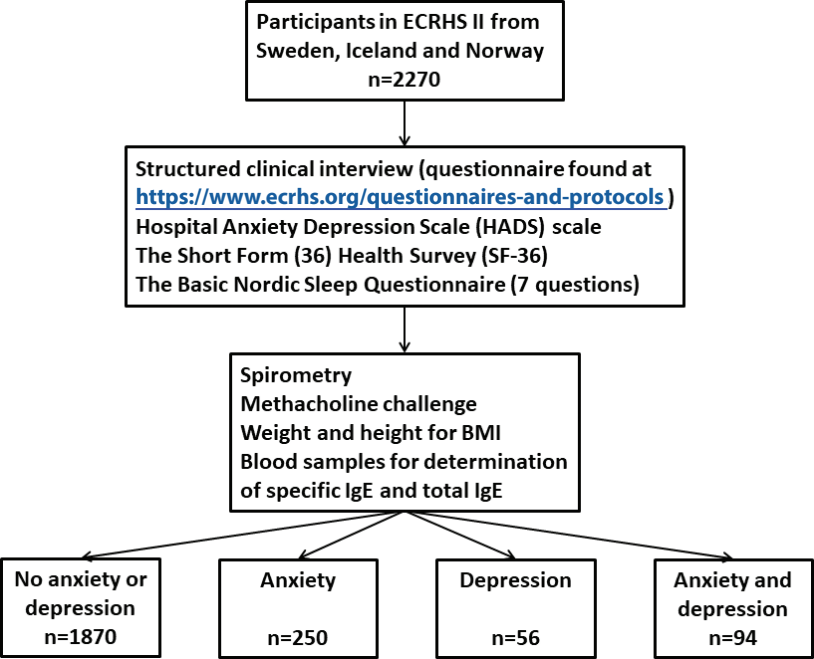
Study design.

ECRHS I (21) was a multicentre study performed at 48 study centres in 1990–1993. The ECRHS II was a follow-up study, performed at 29 centres in 14 countries in 1999–2002 and comprising the participants in the second stage of ECRHS. In the recruitment phase, a postal questionnaire was sent to subjects aged 20–44 years randomly selected from the general population. The questionnaire contained items about asthma symptoms and exacerbation history in the last 12 months, current medication and allergic conditions such as nasal symptoms and hay fever. The response rate of ECRHS I was 84%. From those who responded to the postal questionnaire, participants were either selected from a random sample or a symptomatic sample and were invited for more detailed investigation that included an interviewer-administered questionnaire and lung function testing. The questionnaires and clinical investigations were repeated in the next follow-up ECRHS-II (22). The present analyses include 2,705 subjects from centres in Sweden, Iceland and Norway, who participated in the ECRHS II. Hospital Anxiety and Depression Scale (HADS) was available for 2,270 subjects, Figure 1. The response rate of ECRHS II in the centres included in this analysis was 81%.

### Questionnaires

#### Structured clinical interview

The screening questionnaire and the questionnaire used in the structured interview are based on the International Union against Tuberculosis and Lung Disease (IUTLD) questionnaire (23). Each participant took part in a structured clinical interview including questions on the presence of asthma, respiratory symptoms and therapy. The full questionnaires can be found at https://www.ecrhs.org/questionnaires-and-protocols.

### Short Form Health Survey (SF-36)

HRQL was assessed with the generic SF-36 Health Survey measuring eight domains of health: physical function (PF), role physical (RP), bodily pain (BP), general health (GH), vitality (VT), social function (SF), role-emotional (RE) and mental health (MH). Additional summary measurements, the Physical Component Summary (PCS) and Mental Component Summary (MCS) are based on the eight domains of the SF-36. The Swedish version of the SF-36 questionnaire was adapted from a pre-existing questionnaire (24). The questions were tested for comprehensibility and translated, with a back-translation into English. For each health measurement, a raw score is transformed to a percentage allocation between 0 and 100 (0 = worst possible health status and 100 = best imaginable health status). The two summary indices, the PCS and MCS, are translated using the method proposed by the developers (25). The PCS and MCS do not vary between 0 and 100 but have a narrower range. The PCS varies between 0 and 76, whilst the MCS varies between –5 and 78. The PCS and MCS were calculated, with higher values indicating better health status. The minimal clinically important difference has been calculated as 3 for the PCS and 2 for the MCS (26).

### Asthma score

The ECRHS questionnaire was adapted from a pre-existing questionnaire (24). The questions were tested for comprehensibility and translated, with a back-translation into English. The creation and cross-sectional performance of the asthma score have been reported elsewhere (27, 28). In brief, in the ECRHS questionnaire, there are 12 main questions on asthma symptoms, combined to produce an asthma score consisting of the sum of positive answers to eight of the 12 questions (27, 28). The score used here is a simplification of that score. Three questions including the term ‘asthma’ were subsequently deleted in order to avoid differences in diagnostic practices that might contribute to diagnostic bias. The asthma score without questions with the term ‘asthma’ showed almost the same internal consistency as the score with eight questions, where the rank correlation between the two scores was *r* < 0.95. The final asthma score used in the analyses consisted of the sum of the positive answers to five items, i.e. the score ranges from 0 to 5 (28). The items were:

1. Wheezing in the last 12 months
2. Woken up by a feeling of chest tightness in the last 12 months
3. Attack of shortness of breath at rest in the last 12 months
4. Attack of shortness of breath after exercise in the last 12 months
5. Woken by an attack of shortness of breath in the last 12 months.

### HADS questionnaire

Participants in Sweden, Iceland and Norway were also asked to fill out the self-reported HADS (29, 30). The HADS is a 14-item scale that generates ordinal data. Seven of the items relate to anxiety and seven relate to depression. Each item is rated on a four-point scale, 0 – not at all, 1 – sometimes, 2 – often, 3 – all the time, giving maximum subscale scores of 21 for symptoms of anxiety and depression, respectively. The HADS questionnaire produces clinically meaningful results as a psychological screening tool, in clinical group comparisons and correlation studies, with several aspects of disease and quality of life (31). In the validation of the questionnaire, a score of 0–7 on the two subscales has been found to discriminate non-cases from doubtful cases with a score of 8–10 and scores of 11 or more for definite cases of anxiety and depression, respectively (30). In the present study, we combined the HADS depression and anxiety scale in order to obtain a combined measurement of psychological status (32, 33).

### Insomnia symptoms

The seven questions on sleep disorders were derived from the Basic Nordic Sleep Questionnaire (34) and had been used previously in the Respiratory Health in Northern Europe (RHINE) study (35). Participants were asked to estimate the frequency of different symptoms during the last few months on a five-point scale: 1, never; 2, less than once a week; 3, 1–2 nights per week; 4, 3–5 nights per week; 5, almost every night (29). The aspects of insomnia symptoms were assessed: difficulty initiating sleep (DIS), difficulty maintaining sleep (DMS) and early morning awakening (EMA) (35). A sleep disturbance score was constructed as the maximum value of these three questions divided by 3, and the score, thus, ranges from 1 to 5.

### Smoking history

Smoking history was investigated by asking subjects whether they were current smokers, smokers who had quit smoking between ECRHS I and II, ex-smokers (stopped smoking before ECRHS I) or never-smokers.

### Clinical measurements

#### Spirometry

FEV_1_ (forced expiratory volume in 1s) was measured using a dry rolling seal spirometry system (Sensor medics 2130, Sensor medics, Anaheim, CA, USA). Up to five technically acceptable blows were determined. The results were expressed as a percentage of predicted using the European Coal and Steel Community (36).

#### Body mass index

Body mass index (BMI) was calculated as weight in kilograms divided by the square of height in meters.

#### Atopy

Blood samples were collected for the measurement of serum-specific IgE and total IgE using the Pharmacia CAP System (Pharmacia Diagnostics, Uppsala, Sweden). Serum samples were stored at –20°C and then transferred to a centralised laboratory where they were tested for specific IgE to house dust mite, grass, cat and *Cladosporium*. Atopy was defined as having IgE (≥0.35 kU/L) against at least one of the investigated allergens (37).

### Statistical analysis

For continuous variables, differences between more than two groups were calculated with one-way analysis of variance (ANOVA) and Kruskal Wallis-test to compare differences when the dependent variable was not normally distributed. For categorical variables, differences between groups were compared with the *χ* ^2^-test.

The sample was divided in three equally large groups based on the physical (PCS) and mental health (MCS) component scores.

Associations between the HADS and HRQL with HRQL as the dependent variable were estimated using quantile regression, where the median was modelled. Quantile regression makes fewer assumptions than ordinary least squares regression but at the expense of lower power. Two models were fitted: one for the PCS and another one for the MCS. Initially, additive models were fitted using restricted cubic splines with four degrees of freedom (d.f.) for all continuous variables. Based on the importance of the variables, as judged by the variables’ *χ* ^2^ – d.f., and blinded to the tests of association, the degrees of freedom were adjusted, so that more important variables had more d.f. allocated to them. The results were adjusted for sex, age, smoking habits, BMI, atopy, FEV1 predicted, asthma symptoms score, sleep score and HADS.

With the additive models in place, three interactions were added: the HADS and sex, the HADS and atopy, and the HADS and the number of asthma symptoms. All interaction terms were either kept or deleted based on a simultaneous test of association. A *P*-value of < 0.05 was considered to be statistically significant.

### Ethical approval

Ethical approval from each centre’s regional research ethical committee was obtained.

## Results

For all participants, the mean value of PCS was 49.1 (SD 10.7) and the median 52.6 (IQ range 46.3; 56.1). For MCS, the mean value is 51.9 (SD 10.2) and the median 54.9 (IQ range 48.2; 58.4). The participants had a slightly higher mean age (33.6 (SD 7.1) vs. 33.0 (SD 7.1) years) and were more likely to be current smokers in ECRHS I than those who only participated in ECRHS I. There was no difference concerning sex distribution or the prevalence of ever asthma between those who did or did not participate.

The characteristics of the sample divided in three equally large groups are presented in Table 1. Participants with a low PCS and low MCS were more often women and were more likely to be current smokers. They also had more asthma symptoms, a higher HADS score and a higher sleep disturbance score. A low PCS was more common in older participants, whilst the opposite was found for a low MCS. Participants with a low PCS had a higher mean BMI and a lower FEV_1_. There was no significant association between atopy and the PCS or MCS.

**Table 1:** Characteristics of the sample (*n* = 2,705) divided in three equally large groups, per cent or median (IQ range) in the participants, divided by tertials according to the physical component score (PCS) and the mental component score (MCS), respectively.

	PCS			*P*-value	MCS			*P*-value
	<49.1	49.1–<55.0	55.0–70.1		<51.2	51.2–<57.5	57.5–78.4	
Number	902	902	901		902	902	901	
Males, %	41	52	49	**<0.001** ^^^	41	48	51	**<0.001**
Age, mean (±SD)	44 (±7)	42 (±7)	41 (±7)	**<0.001** *	42 (±7)	42 (±7)	43 (±7)	**<0.001**
Smoking habits, %								
None	41	42	49	**<0.001** ^^^	39	46	46	**0.003**
Ex	18	21	20		19	19	21	
Quitter	13	13	13		15	12	12	
Current	28	24	18		27	22	21	
BMI, mean (±SD)	27 (±5)	26 (±4)	25 (±4)	<0.001*	26 (±4)	26 (±4)	26 (±4)	0.219
Atopy, %	28	27	29	0.680^^^	25	29	30	0.080
FEV_1_ pred. mean (±SD)	101 (±15)	103 (±14)	105 (±12)	<0.001*	103 (±14)	103 (±13)	103 (±14)	0.719
Number of asthma symptoms, %								
0	44	64	76	**<0.001** ^^^	53	64	66	**<0.001**
1	23	20	14		20	19	18	
2	16	10	6		12	10	9	
3	9	3	3		7	4	3	
4	6	2	1		5	2	3	
5	3	1	0		2	1	1	
Sleep disturbance score, %								
1	14	27	29	**<0.001** ^^^	12	22	36	**<0.001**
2	27	35	37		29	38	33	
3	20	17	17		21	18	15	
4	16	10	9		16	12	7	
5	22	11	9		22	10	10	
HADS, mean (±SD)	10 (±6)	7 (±4)	7 (±5)	<0.001*	12 (±6)	7 (±3)	5 (±3)	**<0.001**
PCS, mean (±SD)	–	–	–		49 (±12)	51 (±9)	48 (±11	**<0.001**
MCS, mean (±SD)	52 (±11)	53 (±8)	51 (±11)	<0.001*				

^ Chi-square test.

* ANOVA Kruskal–Wallis test.

PCS = physical component score; MCS = mental component score; HADS = Hospital Anxiety and Depression Scale; BMI = body mass index; FEV = forced expiratory volume.

When controlled for confounding factors (Table 2), older age and a higher BMI were associated with a lower PCS, whilst the opposite was found for the MCS. Current smoking was associated with a lower MCS. The MCS increased with FEV1% predicted, whilst a higher asthma score was related to a lower PCS. Female gender, a higher HADS and sleep disturbance score were all related to both a reduced PCS and a reduced MCS.

**Table 2:** Associations presented as partial (adjusted) effect estimates with 95% confidence intervals for an interquartile range increase in continuous variables and a comparison with a reference category for categorical variables.

	PCS		*P*-value	MCS		*P*-value
	Effect	95% CI		Effect	95% CI	
Males	0.83	(0.14; 1.52)	0.031	0.58	(0.04; 1.11)	0.034
Age	–1.15	(–1.69; –0.62)	**<0.001**	1.21	(0.71; 1.70)	**<0.001**
Smoking habits						
None	Ref		0.255	Ref		0.021
Ex	0.61	(–0.04; 1.26)		0.05	(–0.62; 0.71)	
Quitter	0.43	(–0.67; 1.54)		–0.77	(–1.70; 0.16)	
Current	–0.01	(–0.79; 0.78)		–0.93	(–1.62; –0.24)	
BMI	–1.23	(–1.67; –0.80)	**<0.001**	0.36	(0.04; 0.68)	0.025
Atopy	0.45	(–0.12; 1.03)	0.122	–0.02	(–0.69; 0.66)	0.965
FEV_1_ predicted	0.20	(–0.11; 0.51)	0.200	0.70	(0.30; 1.09)	**<0.001**
Number of asthma symptoms						
0	Ref		**<0.001**	Ref		0.066
1	–2.21	(–3.32; –1.09)		0.18	(–0.63; 0.98)	
2	–2.98	(–5.04; –0.92)		0.14	(–0.54; 0.82)	
3	–3.60	(–5.70; –1.51)		–1.03	(–3.59; 1.53)	
4	–6.83	(–10.02; –3.65)		2.38	(0.85; 3.91)	
5	–5.87	(–7.04; –4.71)		0.05	(–2.99; 3.10)	
Sleep score						
1	Ref		**<0.001**	Ref		**<0.001**
2	–0.35	(–0.97; 0.26)		–1.29	(–1.92; –0.66)	
3	–0.90	(–1.94; 0.14)		–1.56	(–2.29; –0.83)	
4	–2.31	(–3.51; –1.11)		–1.84	(–2.91; –0.77)	
5	–2.83	(–3.94; –1.72)		–1.66	(–2.94; –0.37)	
HADS	–1.10	(–1.95; –0.25)	**<0.001**	–5.44	(–5.93; –4.96)	**<0.001**

All the other variables in the table were set at their medians or most frequent categories. *P*-values are from a joint test of all regression coefficients associated with a particular variable, including non-linear and/or interaction terms (*n* = 1,974). PCS = physical component score; MCS = mental component score; HADS = Hospital Anxiety and Depression Scale; BMI = body mass index; FEV = forced expiratory volume.

**Figure 2:**
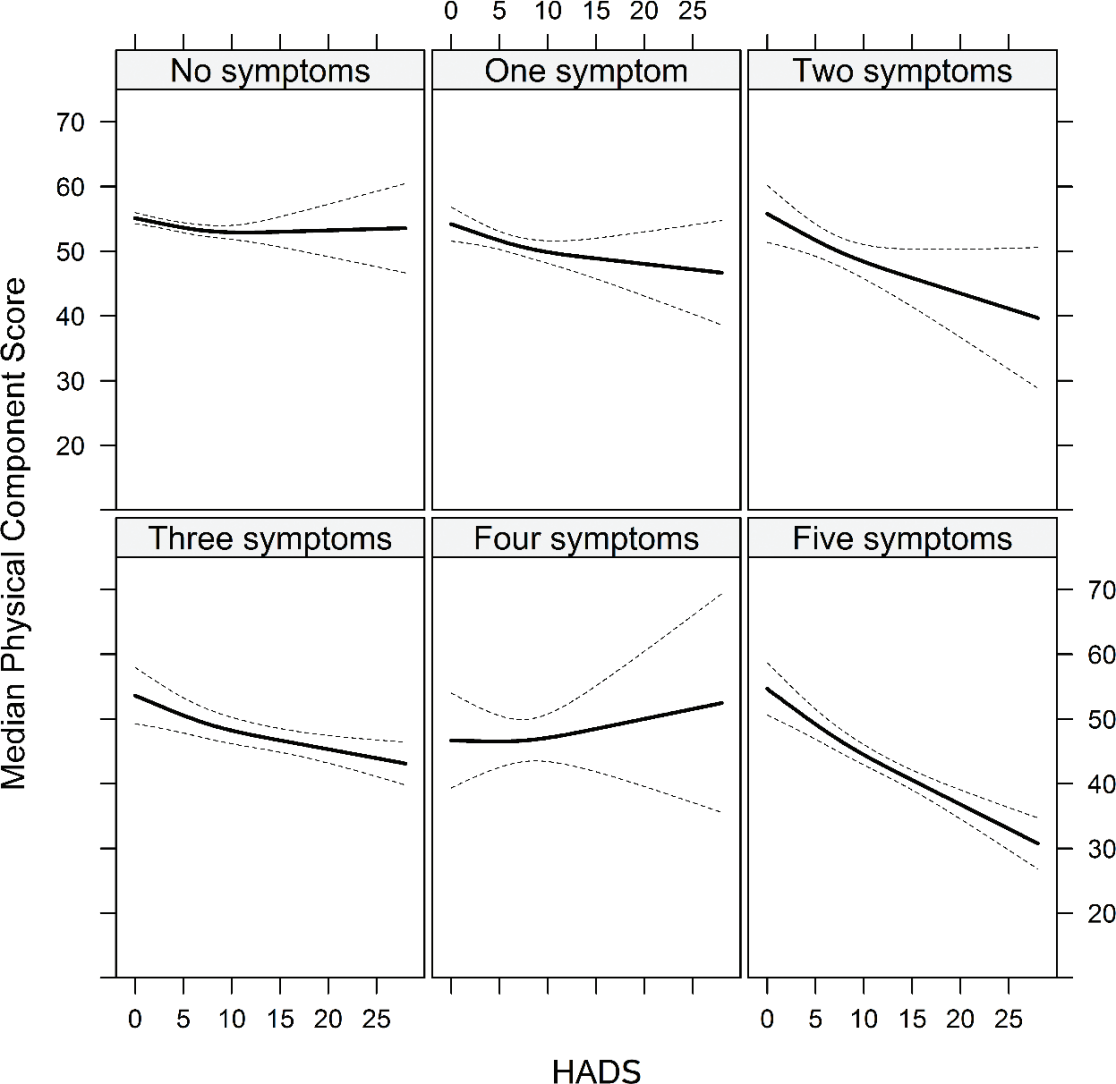
Associations between the HADS and the PCS, by the number of asthma symptoms. There was a clear indication of interactions in the PCS model (*χ*^2^ = 45.56 on 7 d.f., *P*-value < 0.0001) with the number of asthma symptoms modifying the association between the HADS and PCS (*χ*^2^ = 24.57 on 5 d.f., *P*-value = 0.0002). No corresponding interaction was found amongst the HADS, MCS and asthma symptoms. HADS = Hospital Anxiety and Depression Scale; PCS = physical component score; MCS = Mental Component Summary.

The adjusted associations between the HADS and PCS stratified by the number of asthma symptoms are shown in Figure 2. The PCS decreased with the number of asthma symptoms. There was an interaction in the PCS model (*χ*^2^ = 45.56 on 7 d.f., *P*-value < 0.0001 for the overall test of all interaction coefficients), with the number of asthma symptoms modifying the association between the HADS and PCS (*χ*^2^ = 24.57 on 5 d.f., *P*-value = 0.0002). No interactions could be seen in the MCS model (*χ*^2^ = 3.83 on 7 d.f., *P*-value = 0.7995).

The association between the HADS and PCS for individuals without asthma symptoms was almost flat. As the number of asthma symptoms increases, the regression line slopes downwards, indicating that an increased HADS was associated with a lower PCS, with the association being strongest for individuals with five asthma symptoms. The regression line changes sign for individuals with four asthma symptoms, indicating that an increased HADS is associated with an increased PCS in those individuals.

## Discussion

HRQL was independently related to the HADS, insomnia and number of asthma symptoms. There was an interaction between the number of asthma symptoms and the HADS, with the strongest association between the HADS and PCS (physical health component) in those with the highest asthma symptom score. The difference between those with a high asthma score and those with a lower score was well above what has been reported as the minimally clinically important difference for PCS, and the same was true for the association between HAD and MCS (26). High age and high BMI were associated with low PCS scores, whilst the opposite was found for the MCS. Current smoking and a low FEV1 were associated with a low MCS (mental health component), whilst a higher asthma score was related to a low PCS. Female gender, a high HADS score and a higher sleep disturbance score were related to both a low PCS and a low MCS. To our knowledge, this is the first study of HRQL using the generic instrument, the SF-36, an asthma symptom scale, as well as measuring the HADS and insomnia.

The result that a higher asthma symptom score was related to a low PCS is in line with a previous study, showing that the severity of respiratory symptoms was an independent determinant of poorer HRQL (38). The finding that a high HADS and a sleep disturbance score were both related to a low PCS, and a low MCS illustrates that HRQL is even more impaired in asthmatics with coexisting symptoms of anxiety and depression or insomnia and corresponds to earlier findings (39).

The MCS was strongly associated with symptoms of depression and anxiety. Several studies have found correlations between a poor level of asthma control and both symptoms of anxiety and depression. Moreover, the presence of symptoms of anxiety and depression has been associated with frequent asthma exacerbations and higher healthcare utilisation (3, 40).

One explanation of the strong association between HRQL and symptoms of anxiety/depression might be a higher prevalence of uncontrolled asthma. It has been shown that asthmatic subjects with anxiety and depression are less likely to adhere to medication regimens than asthmatics without depression (3, 41), and a lack of asthma control has been associated with impaired HRQL (4, 42–44).

Our results indicate that symptoms of anxiety and depression are of less importance for the physical component of quality of life in subjects with well-controlled asthma. However, symptoms of anxiety and depression appear to be of great importance in asthmatics with uncontrolled or difficult-to-treat asthma.

We found that insomnia was associated with a lower PCS. It has previously been shown that insomnia has a negative impact on several domains of HRQL, such as vitality and energy, but this also extends to other aspects of mental, social and physical functioning (45). Previous studies have reported that insomnia is common amongst asthmatics, that lifestyle factors such as smoking and obesity are risk factors for insomnia in asthma and that symptoms of anxiety or depression with concurrent insomnia are important predictors of asthma-related quality of life (16, 39, 46).

In a Canadian study, insomnia was associated with increased psychological symptomatology and perceived stress, as well as impaired HRQL (47). In a longitudinal Norwegian study, insomnia was a risk factor for a range of both physical and mental conditions (48). We also found that female gender, and high HADS and sleep disturbance scores were related to both a low PCS and a low MCS. This corresponds to a previous study, showing that women with asthma had a higher prevalence of symptoms of anxiety, insomnia and daytime sleepiness than men (29).

Our finding that older subjects had a lower PCS and a higher MCS confirms the result from previous studies, showing that advanced age is an independent negative risk factor for quality of life in asthmatics (6, 49). The same studies demonstrated that impaired lung function is a negative risk factor for quality of life in asthmatics. In addition, in the present study, a low FEV1 was associated with a low MCS.

An increased BMI was associated with an increased MCS but a decreased PCS. Obesity is over-represented in asthma and has previously been shown to correlate with poorer asthma control, more frequent exacerbations and poorer quality of life (6, 50–53). Obesity may affect ventilation capacity, resulting in worse symptoms, less ability to exercise and subsequently the mood of the patient.

Current smokers had poorer HRQL in both the PCS and MCS, whilst ex-smokers had a higher PCS but a lower MCS. This is in line with the results of previous studies, showing poorer quality of life (6), poorer asthma control and a higher prevalence of symptoms of anxiety amongst smoking asthmatics (54).

The strength of the present analysis lies in the large, well-characterised population with asthma in the framework of ECRHS II and a standardised validated generic HRQL questionnaire (SF-36). One limitation of generic instruments versus asthma-specific quality of life instruments is that the SF-36 has more valid measurements than asthma-specific quality of life instruments when applied to individuals with asthma from the general population (12). The asthma score in this study presents an option to analyse asthma symptoms as a continuous variable that increases the power of the analyses, with good predictive ability against outcomes related to asthma and a good ability to detect risk factors.

Although the study benefits from validated, well-characterised measurements of HRQL, symptoms of insomnia, anxiety and depression, it is not without its limitations. Co-morbidity such as gastro-oesophageal reflux, chronic rhinosinusitis and obstructive sleep apnoea, which correlate to both asthma control and insomnia symptoms, could, for example, not be assessed.

Another potential limitation is the inconsistent reliability of self-reported conditions, such as asthma, insomnia and psychological function, although the reliability of some self-reported diagnoses, e.g. diabetes, appears to be fairly good (48). Another weakness of the study is small numbers in the four-symptom asthma score group. Surprisingly, the regression line in Figure 2 changed direction for individuals with four asthma symptoms, indicating that an increased HADS is associated with an increased PCS in those individuals. The most likely explanation might be the small sample size in this group. Another limitation is the cross-sectional study design.

Our findings indicate that it is important to address psychological symptoms and psychiatric disorders in patients with asthma in order to improve quality of life, especially in asthma that is not well controlled. One reason for our findings could be that patients with symptoms of anxiety and depression have more difficulty following treatment than asthmatics without psychological problems, resulting in less well-controlled asthma in subjects with anxiety and depression due to sleep disturbances, for example. Subjects with asthma should be supported to follow prescriptions in order to achieve good asthma control. This support is probably more important in patients with symptoms of anxiety and depression. Smoking and obesity also need to be taken into account. In a recent Spanish study, patients with asthma under standardised asthma care supervised by an asthma specialist exhibited significantly improved levels of anxiety and depression symptoms, obtained better asthma control and an improvement in FEV_1_ (13).

In conclusion, HRQL was independently related to psychological status, insomnia and asthma control. There was an interaction between the number of asthma symptoms and psychological status, with the strongest association between psychological status and the PCS in those with the highest asthma symptom score. The findings suggest that treatment of anxiety and depression may be important in patients with many asthma symptoms in order to increase HRQL. In fact, these studies have already started showing positive results (55). However, future research needs to investigate the impact of different behavioural interventions to increase HRQL in subjects with the complex mixture of asthma symptoms and insomnia problems.

## ORCID

Anna Rask-Andersen https://orcid.org/0000-0001-9412-6242

Mai Leander https://orcid.org/0000-0002-3944-8633

Fredrik Sundbom https://orcid.org/0000-0001-6627-9735

Erik Lampa https://orcid.org/0000-0002-3268-8810

Anna Oudin https://orcid.org/0000-0002-9876-0627

Benedicte Leynaert https://orcid.org/0000-0001-5045-2492

Cecilie Svanes https://orcid.org/0000-0001-8512-5192

Thorarinn Gislason https://orcid.org/0000-0002-6773-9876

Kjell Torén https://orcid.org/0000-0001-8509-7603

Christer Janson https://orcid.org/0000-0001-5093-6980
